# Approaches and Perspectives for Development of African Swine Fever Virus Vaccines

**DOI:** 10.3390/vaccines5040035

**Published:** 2017-10-07

**Authors:** Marisa Arias, Ana de la Torre, Linda Dixon, Carmina Gallardo, Ferran Jori, Alberto Laddomada, Carlos Martins, R. Michael Parkhouse, Yolanda Revilla, Fernando Rodriguez, Jose-Manuel Sanchez-Vizcaino

**Affiliations:** 1 European Union Reference Laboratory for ASF, Centro de Investigación en Sanidad Animal (INIA-CISA), 28015 Madrid, Spain; torre@inia.es (A.D.L.T.); gallardo@inia.es (C.G.); 2 The Pirbright Institute (TPI), Surrey GU24 0NF, UK; linda.dixon@pirbright.ac.uk; 3 ASTRE, University of Montpellier, CIRAD, INRA, F-34398 Montpellier, France ferran.jori@cirad.fr; 4 Istituto Zooprofilattico Sperimentale della Sardegna (IZS-Sardegna), 07100 Sassari, Sardinia, Italy; albertolad@live.com; 5 Faculdade de Medicina Veterinária (FMV-ULisboa), 1300-477 Lisbon, Portugal; cmartins@fmv.ulisboa.pt; 6 Instituto Gulbenkian de Ciência (IGC), Rua Quinta Grande 6, 2780-156 Oeiras, Portugal; parkhous@igc.gulbenkian.pt; 7 Centro de Biología Molecular Severo Ochoa (CBMSO-CSIC-UAM), C/ Nicolás Cabrera nº 1, Campus de Cantoblanco, 28049 Madrid, Spain; yrevilla@cbm.csic.es; 8 Institute for Research and Technology Food and Agriculture (IRTA), Centre de Recerca en Sanitat Animal (CReSA, IRTA-UAB), Campus de la Universitat Autònoma de Barcelona, 08193 Bellaterra, Spain; fernando.rodriguez@irta.es; 9 OIE Reference Laboratory for ASF, Centro de Vigilancia Sanitaria Veterinaria (VISAVET), Universidad Complutense de Madrid, Avda. Puerta del Hierro, 28040 Madrid, Spain; jmvizcaino@ucm.es

**Keywords:** African swine fever, vaccine, immunology, vaccine gaps

## Abstract

African swine fever (ASF) is a complex disease of swine, caused by a large DNA virus belonging to the family Asfarviridae. The disease shows variable clinical signs, with high case fatality rates, up to 100%, in the acute forms. ASF is currently present in Africa and Europe where it circulates in different scenarios causing a high socio-economic impact. In most affected regions, control has not been effective in part due to lack of a vaccine. The availability of an effective and safe ASFV vaccines would support and enforce control–eradication strategies. Therefore, work leading to the rational development of protective ASF vaccines is a high priority. Several factors have hindered vaccine development, including the complexity of the ASF virus particle and the large number of proteins encoded by its genome. Many of these virus proteins inhibit the host’s immune system thus facilitating virus replication and persistence. We review previous work aimed at understanding ASFV–host interactions, including mechanisms of protective immunity, and approaches for vaccine development. These include live attenuated vaccines, and “subunit” vaccines, based on DNA, proteins, or virus vectors. In the shorter to medium term, live attenuated vaccines are the most promising and best positioned candidates. Gaps and future research directions are evaluated.

## 1. Introduction

African swine fever virus (ASFV) is the cause of African swine fever (ASF), an important disease affecting both wild and domestic swine of all breeds and ages. In domestic pigs and wild boar, ASF is associated with a number of clinical manifestations including a hyper-acute or acute disease with very high mortality rates [1,2,3]. Subacute forms, which result in reduced mortality of 30 to 70%, as well as sub-clinical or chronic disease forms which can result in very low if any mortality, have also been described [4]. Infection has been long established in wild suids in Africa, including warthogs and bushpigs, causing long unapparent infections. These mammalian hosts, together with invertebrate Ornithodoros ticks, can act as natural ASFV reservoirs in some areas of the sub-Saharan Africa, acting as a permanent source of ASF virus for domestic pigs. ASFV is commonly transmitted when unexposed pig populations (domestic or wild) have direct contact with blood, excretions, secretions, meat, or carcasses from infected animals or indirect contact with infected products. ASF notification is mandatory because of the great sanitary and socio-economic impact on the pig industry, which include bans on international trade in live animals and swine products.

ASFV is the unique member of the family Asfarviridae. The virus genome is double-stranded DNA and virions have a characteristic icosahedral capsid assembled on an internal membrane and surrounding a nucleoprotein core. An additional membrane is derived from the plasma membrane as virus buds from cells, both intracellular [5,6,7]. Intracellular mature and extracellular enveloped forms of the virion are infectious [8,9]. The DNA genome shows significant variations in length from 170 to 193 kbp depending on the isolate, thus coding for between 150 and 167 open reading frames (ORFs) [10,11,12,13,14], specifying the 54 structural proteins of the ASFV particle and more than 100 infection proteins [15]. On the basis of molecular genotyping, 23 distinct genotypes of ASFV have been described to date. All genotypes are present in sub-Saharan Africa, while only genotype I and genotype II have spread outside Africa. ASFV genotype I spread to the Iberian Peninsula in 1957 and 1960, with later incursions into some other European countries, the Caribbean, and Brazil, and still remaining in Sardinia. Genotype II spread from East Africa to the Caucasus region in 2007 and then spread rapidly and widely throughout the Russian Federation and a number of countries in Eastern Europe.

ASF epidemiology is complex and depends on the characteristics of the virus circulating, the presence of wild and domestic hosts and reservoirs of infection, and on environmental, social, and cultural factors. After years in some regions, mortality rates of pigs have been observed to decline over time, to become subacute, chronic, or subclinical forms of the disease caused by the emergence of moderate and low virulent virus isolates. These variable clinical forms can be difficult to recognize and may persist in surviving pigs, providing a potential reservoir for infection. Survivors from sub-acute infections were shown to shed virus from their oropharynx for at least 70 days [16,17,18]. Virus can also be isolated from porcine tissues for up to 180 days post infection [19,20,21,22]. Thus, contaminated, uncooked pig meat swill fed to pigs and movement of infected animals are common routes for virus transmission.

ASF is present in twenty-eight sub-Saharan African countries, and in Sardinia, Italy since 1978 [23,24]. Following an increased epidemic in sub-Saharan countries, in 2007 ASFV spread far beyond its historical limits, first to Georgia [25,26] and now reported in the Caucasus region, north-west and central Russian Federation, Belarus, Moldova, and some eastern EU countries (Lithuania, Poland Estonia, Latvia, Czech Republic, and Romania). Effective control of ASF has not been achieved in Africa or Europe, providing a serious threat to the global pig industry.

No vaccine is available against ASF. Prevention, control, and eradication measures are mainly based on early detection by efficient laboratory diagnosis and on the implementation of strict sanitary measures [27]. Vaccination to contain viral infections in livestock is generally regarded as the most cost efficient measure if available. These facts, combined with the re-emergence of ASF in the European Continent have increased interest in the development of a vaccine against ASF as an additional control tool. The availability of effective and safe ASF vaccines would improve ASF disease control and eradication programs thus reducing economic losses in the endemic regions. Work leading to the rational development of a protective ASF vaccine is therefore a priority. This review describes previous and current strategies to develop effective and safe vaccines for ASFV. Future prospects are also evaluated.

## 2. Immune Response against ASFV

Understanding the intricacies of protective immunity to ASFV is a key issue for vaccine development. However, this is still poorly characterized. Nevertheless, it is clear that pigs that recover from infection are resistant to challenge against some ASFV isolates, indicating that these animals can develop a protective immune response [28,29,30,31,32,33,34,35,36,37]. However, the complexity of ASFV, a virus encoding more than 160 different polypeptides, many of them specialized in evading different aspects of the immune system [38], together with the variability of the virus isolates so far identified has complicated this task.

Attenuated ASF viruses obtained by tissue-culture adaptation conferred solid protection against parental virulent viruses, but did not confer protection against heterologous viruses, including against ASFV isolates found in close geographical ad temporal proximity [31,39]. Similarly, several investigations have shown that animals that survive infection with less virulent isolates can be protected against challenge with related virulent viruses [28,40,41]. The extent of cross-protection against different genotypes has been little studied, although there are reports of cross-protection between certain genotypes [36,42,43,44,45]. Currently, the virus antigens important for cross-protection have not been fully characterized, although the virus CD2v-like protein has been suggested to be a candidate [42,45,46,47].

Considerable difficulties have been encountered while searching for immune correlates of protection. Nevertheless, it seems clear that the protective immune response includes both cellular and serological immunity [32,33,35,37,48,49]. Some findings, such as the lack of fully neutralizing antibodies, remain controversial [50]. However, evidence for some role of antibody-mediated immunity in protection has been obtained. Thus, passive transfer of sera from ASFV-infected and recovered pigs partially protected pigs against homologous (parental) ASFV challenge infection and the potential fatal consequences of infection by delaying the onset of the ASF clinical signs and reducing the levels of viremia [51,52,53,54,55].

A variety of in vivo and in vitro studies indicate potentially protective roles of antibodies by additional mechanisms including complement mediated cell lysis or antibody-dependent cell-mediated cytotoxicity (ADCC) [37,50,56,57,58,59,60,61]. An interesting correlation has been established between the presence of haemadsorption (HAD) inhibitory antibodies in a serum with its capacity to inhibit the infection of ASFV in vitro and to partially protect against ASFV challenge in vivo [42,62].

Evidence also indicates a key role for NK cells [63] and specific T cell responses in protection [33,35,63]. Using pigs recovered from experimental infection with the naturally attenuated ASFV-isolate NHVNon Haemadsorbing Portugal 68 (NHP68) as an experimental model, the key protective role of the CD8-T cell subset in virus elimination as a result of cytotoxic activity was observed [33]. Antibody depletion of the CD8^+^ cell population abrogated the protection induced by the natural attenuated strain OURT88/3, demonstrating an essential role for this cell subset in protection [35]. In conclusion, evidence available indicates that immune protection involves antibody-mediated and cell-mediated mechanisms. 

## 3. Proteins Involved in Immune Evasion and Virulence

A better understanding of virus–host interactions is required for vaccine development. Important objectives that would help to define rational vaccine approaches are the identification of host cell receptors and the virus proteins interacting with these, improved knowledge on virus mechanisms to overcome the protective host barriers inhibiting virus replication, and of the host immune mechanisms involved in protection.

The ASFV targets are mainly macrophages. These cells have an extremely important role in activating and orchestrating the immune response against virus infections [64,65]. ASFV uses a number of strategies to evade the host’s defense systems, including innate and intrinsic immune mechanisms such as type I interferon (IFN) responses, apoptosis, inflammation, and the activation of specific target genes during ASFV infection, [66,67]. Identifying the key genes and their corresponding proteins mediating such processes is of great importance in understanding virus–host interactions and is fundamental for the design an effective live-attenuated vaccine.

Some progress towards the characterization of such virus “host evasion” genes has been made. For example, the non-essential A238Lp, which prevents transcriptional activation of host immune response genes by inhibiting host transcription factors. Several virus proteins are known to regulate and inhibit programmed cell death pathways at early times of infection. These include the non-essential proteins A179Lp, a Bcl-2 family member, A224Lp, Inhibitor of Apoptosis (IAP) family member, and C-type lectin EP153Rp. This allows virus replication and production of progeny virus to proceed. In contrast, other proteins such as the essential structural protein p54/E183Lp, may modulate the production of virus particles and the mechanisms of release by inducing apoptosis at late times of infection. The DP71Lp protein recruits protein phosphatase 1 to dephosphorylate translation initiation factor eIF2 and restore global protein synthesis. Several proteins are involved in inhibiting the induction of IFN including those coded for by the multigene family (MGF) 360 and 505 genes and the I329L, K205R, and A276R genes [67,68,69,70,71,72,73,74,75,76,77,78,79,80,81,82,83,84]. The I329Lp protein has been characterized as a glycoprotein localizing in the host cell surface membranes and was the first ASFV protein demonstrated to inhibit the IFN response through the Toll-like Receptor 3 (TLR3) signaling pathway [71]. In addition, I329Lp also inhibits TLR4 signaling. The K205Rp protein was shown to localize in the cytoplasm and to inhibit activation of IFN-β. The A276Rp protein was also identified as an inhibitor of IFN-β activation, and does not appear to target IRF-7. As a consequence of the inhibition of IFNαβ, expression of the hundreds of IFN stimulated genes is inhibited. These have broad functions involved in activating an antiviral state in infected and bystander cells and activating host immune responses. The D96Rp (also referred to as UK) protein is also a potential immune evasion gene, although its mechanism of action is unclear [85]. Other modulatory proteins include the hemmaglutinin CD2v/E402Rp protein that is present on the surface of extracellular virions and inhibits activation of lymphocytes, [86,87,88]. These mentioned genes are good candidate targets for the development of an attenuated gene deletion mutant virus for vaccine development. 

Finally, some essential ASFV proteins have been characterized, including ASFV-Toposoimerase II [89,90,91], a histone-like protein [92], and the ASFV-E2 Ubiquitin conjugating enzyme, opening new avenues to generate effective single-cycle mutant virus vaccines using helper cell lines expressing these essential proteins. Similar approaches have been reported for Bluetongue disease [93,94,95] and African horse sickness [96,97]. Both gene deletion attenuated and replication deficient viruses have the advantage of presenting almost the entire virus repertoire via Major histocompatibility complex (MHC) class I and II to CD8 and CD4 T cells, respectively, thus stimulating both cellular and serological immunity.

## 4. Virus Proteins Important for Inducing Protective Antibody Responses

**Figure 1:**
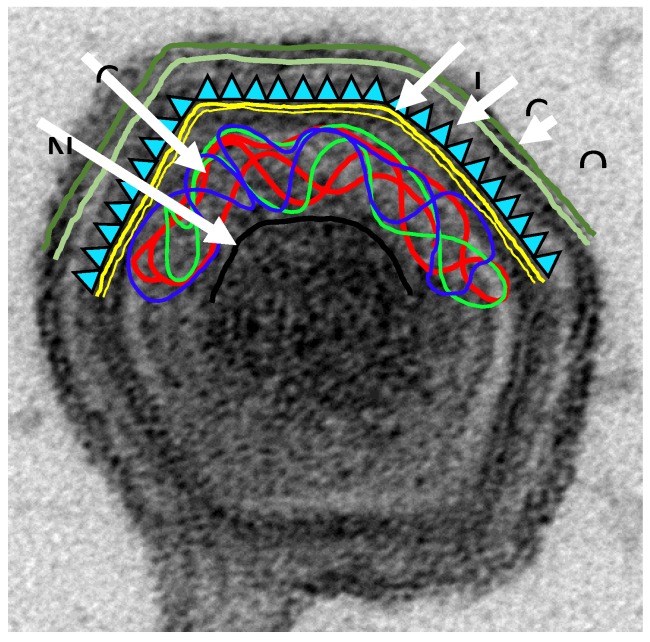
African swine fever virus (ASFV) structure. An electro micrograph of an extracellular ASFV particle budding through the cell plasma membrane is shown. The particle is large (~200 nm) and complex containing more than 50 proteins. Several layers are indicated on the cartoon. The nucleoprotein core (NU) is surrounded by the core shell (CS) and the inner envelope (IE) on which the icosahedral capsid (CA) is assembled. This intracellular mature particle is assembled in cytoplasmic virus factories. The extracellular virus particles gain an additional outer envelope (OE) budding through the cells plasma membrane. The OE contains ASFV proteins, CD2v/EP402Rp, p12/O61Rp and the cellular protein designated p24; the CA contains major protein p72/B646Lp and also E120Rp, B438Lp; the IE contains p17/D117Lp, p54/E183Lp, E248Rp and p12/O61Rp; CS contains the cleavage products of polyproteins pp220/CP2475Lp (p150, p37, p34, p14) and pp62/CP530Rp (p35, p15) and S273Rp; the NU contains p10/K78Rp, 104Lp, proteins and enzymes required to initiate infection including the virus RNA polymerase as well as the virus genome.

Identification of virus proteins which may be targets of antibody mediating neutralization or elimination still merits more investigation. Virus proteins present on the surface of both intracellular mature and extracellular enveloped infectious virions and the surface of infected cells are expected to be important proteins for antibody-mediated protection (see Figure 1). Virus targets for neutralization have been identified including p72/B646Lp, p54/E183Lp, and p30/CP204Lp. Antibodies against p72/B646Lp and p54/E183Lp inhibited virus binding to cells, whereas those against p30/CP204Lp inhibited virus internalization. Other virion proteins present on the surface of intracellular mature or extracellular enveloped virus particles may be targets for neutralization by preventing virus entry or spread. These include the CD2v/EP402R protein, p12,/O61Rp, D117L proteins [42,50,58,98,99,100].

The characterization of cell receptors on pig macrophages is of interest in order to identify virus and host molecules involved in virus entry as targets to inhibit this process. These molecules may also include intracellular host proteins. For example, cellular proteins involved in virus release from endosomes to the cytoplasm or transport within the cytoplasm may be important [101,102]. The CD2v/EP402Rp interaction with the cellular adaptor AP-1 may be involved in movement of the viral particle, and thus have consequences for virulence and immune escape [101].

Antibodies that inhibit virus spread would also be useful. Studies on the mechanisms of viral entry showed that ASFV uses macropinocytosis [103] and other mechanisms, including clathrin mediated endocytosis to enter porcine macrophages [104].

A rational identification of potential protective serological determinants of protective immunity (by screening of a virus expression library with a polyclonal antisera from a domestic pig surviving infection with virulent virus strain) identified fourteen serological immunodeterminants, including virus proteins B602Lp, C44Lp, CP312Rp, E183Lp, K145Rp, and K205Rp, as well as the structural proteins A104Rp, p10/K78Rp, p30/CP204Lp, p54/E183Lp, p72/B646Lp, and the non-structural proteins ribonucleotide reductase (F334Lp, F778Rp), DNA ligase (NP419Lp), and Thymidine kinase (K169Rp) [105].

## 5. Virus Proteins Important for Inducing T Cell Mediated Immunity

There is strong evidence for an important role for specific CD8^+^ T cells in protection [35,106]. By DNA immunization of pigs, partial protection against ASFV was demonstrated in the absence of specific antibodies, correlating with the induction of specific CD8^+^ T cells against the CD2v (hemagglutinin) [107,108]. DNA vaccination with a plasmid library identified multiple cytotoxic T lymphocyte (CTL) epitopes with protective potential [37]. Further work is required to characterize relevant antigenic epitopes, and complications arising from variability of MHC peptide presentation within outbred pig population.

CTL determinants have been described before in the G1340Lp protein [109] and in the ASFV p30/CP204Lp and p72/B646Lp structural proteins [110,111], but their role in protection has not been demonstrated. Identification of ASFV CTL epitopes relevant for protection is a complicated issue due to the heterogeneity of the T cell population [34].

In addition to CD8 T cells, other subsets of T cells might play an important role in protection [37]. A deeper understanding on the role of T cells as well as NK and other cells from the innate immune system should facilitate formulating optimal subunit vaccine formulations in the future.

## 6. Approaches towards ASFV Vaccine Development

ASFV vaccine development has been investigated since the 1960s. Approaches used have included inactivated viruses, recombinant proteins/peptides, viral vectors for antigen delivery, and live-attenuated vaccines. As of yet, none of these experimental approaches have been taken forward for evaluation of their potential for commercial production.

### 6.1. Inactivated Candidate Vaccines

To date, inactivated preparations of ASFV have not conferred protection even in the presence of adjuvants, a not entirely surprising finding if cellular immunity is essential for protection. In addition the possibility of antibody-mediated enhancement of the infection has been observed [112,113,114,115,116]. The complexity of the virus particle which contains more than fifty proteins in several layers and the fact that there are two infectious forms, an intracellular mature and extracellular form, might additionally contribute to this failure in conferring protection since effective virus neutralization is difficult to achieve in primary infections.

### 6.2. Subunit Vaccine Approaches

ASFV encodes up to 167 proteins making it very difficult to select candidate antigens that can induce protection for incorporation in subunit vaccines. As described above, several ASFV proteins have been reported to be targets for virus neutralization and the potential for these proteins to induce protection has been tested.

While co-immunization of pigs with p54 and p30 expressed in baculovirus conferred significant protection against lethal challenge with E75 [98], a combination of p54 + p30 + p72 baculovirus expressed proteins did not protect against lethal challenge with the pathogenic Malawi isolate [100]. These contradictory results might be partially explained by the virus strain used, albeit more recent work with DNA vaccines encoding p54 and p30 did not induce neutralizing antibodies or show any protection against lethal infection with E75 [117]. However, these results are difficult to compare due to the very different nature of protein versus DNA immunization protocols. In a different study, the CD2v/EP402R gene, when expressed in a baculovirus system, induced some degree of protection against a challenge with virulent virus. This correlated with the induction of antibodies that inhibited haemadsorbtion (HAD) and temporarily inhibited infection [62]. Recent evidence indicates that CD2v/EP402R and/or C-type lectin/EP153R proteins may be important for protection against ASFV infection [42].

DNA vaccination has also been used to identify potentially protective antigens. Immunization of pigs with a gene fusion of p30/CP204L and /p54/E183L, with the gene for a single chain variable fragment of a specific antibody against swine leukocyte antigen II, induced ASFV specific T cells. However, neither neutralizing antibodies nor protection against a virulent challenge was reported [107]. Fusion of gene fragment coding for the extracellular domain of HA (CD2v/EP402R) fused to the Lp30/CP204L and p54/E183L genes enhanced both humoral and cellular responses in pigs, without conferring protection. However, fusion of these three ASFV-genes (CD2v/EP402R, p54/E183L and p30/CP204L) to the ubiquitin gene, induced strong CTL responses and conferred partial protection in the absence of specific antibodies. This protection correlated with the proliferation of HA (CD2v/EP402R)-specific CD8^+^ T cells [117]. Further immunization with a DNA expression library containing several other viral ORFs fused to ubiquitin also conferred partial protection against a virulent challenge [108]. Once again, this protection correlated with the induction of ASFV specific T cells and the absence of detectable antibodies, highlighting the role of T cell responses in protection and revealing the existence of multiple ASFV antigens with potential protective capacity. Despite the utility that these strategies might have in the future for dissecting both the immune mechanisms and the ASFV-antigens involved in protection, they are today far from providing the level of protection required to be useful in the field.

Prime-boost strategies have been carried out using combinations of specific ASFV recombinant proteins and DNA but no protection against challenge with Armenia strain was observed despite induction of robust immune responses [118]. Table 1 summarizes the current approaches for development of subunit protein or DNA vaccines.

Immunization of pigs with pools of recombinant adenoviruses expressing individual ASFV proteins and boosted with either the same vectors [119] or with recombinant modified vaccinia Ankara strain (MVA) expressing the same antigens [120] also induced robust cellular and antibody responses although pigs were not challenged with ASFV [119,120].

Further work will be needed in order to identify both the antigens to be included in a potential subunit vaccine and the optimal immune mechanisms to be triggered after vaccination in order to confer solid protection against ASFV. Optimal delivery systems for immunization of pigs also need to be identified. 

### 6.3. Live Attenuated Vaccines (LAVs)

#### 6.3.1. LAVs Obtained from Virulent and Naturally Occurring Low Virulent ASFV Isolates

The use in the field of LAVs produced by the attenuation of naturally occurring virulent strains has been limited to the extensive experience in Portugal and Spain during the early 1960s [121]. At that time, a large number of animals were vaccinated with LAVs in field conditions, in which animals were exposed to multiple infections and re-infections with circulating field strains viruses by different routes and probably including exposure to infected soft ticks. Vaccinations under these conditions led to the appearance of chronic forms of ASF [122]. From the field experiment in Spain, some animals showed chronic clinical signs. These vaccines are not used anymore mainly due to safety problems derived from their inherent infectious nature [123].

Other experimental strategies have involved the immunization of pigs with the naturally attenuated ASFV strains OURT88/3 or NH/P68. Immunized pigs were protected against challenge with related virulent strains [35,40,42,63], and partial cross-protection has been shown against heterologous viruses [42,43,44,45,46]. The protection levels varied from 66% to 100% dependent on the pigs and the challenge virus, as well as the delivery route and administration dose [36,40,43,44,89]. As described for the subunit vaccines, both specific antibodies [53] and specific CD8^+^ T cells [35], seem to play a crucial role in the protection afforded by LAVs. Cross-protection induced by the OURT88/3 isolate against challenge with virulent isolates from different genotypes was correlated with the ability of those isolates to specifically stimulate IFNγ-producing lymphocytes from the immunized pigs [36]. Despite the correlation between the induction of specific T cell responses and protection [36,37,108], this is far from being a confirmed prediction and other immune mechanisms are under investigation to identify key players in protection. However, the attempts using these naturally attenuated ASFV strains as vaccines have so far demonstrated several side-effects, at least at certain doses, since a substantial proportion of the vaccinated pigs developed unacceptable post-vaccination reactions including pneumonia, locomotor disturbances, necrotic foci, abortion, and death. In the best scenario, pigs do not show significant clinical signs with the exception of transient fever and low viremia that coincides with low nasal shedding in some vaccinated pigs [43,44,63].

#### 6.3.2. Recombinant LAVs Obtained from Virulent Viruses

Recombinant ASFVs containing specific deletions of genes, such as the thymidine kinase (TK) gene, could yield non-pathogenic viruses [45,81,124]. In addition, depletion of genes involved in the evasion of the immune response, NL (alternatively named DP71L) gene, and multiple members of multigene families 360 and 505 (MGF 360/505), or genes involved in virus replication or morphogenesis and 9GL (B119L) gene, have resulted in attenuation of virulent ASFV isolates and induction of protective immune responses against virulent parental virus challenge, but with varying levels of residual virulence [125,126,127,128]. However, the effects of gene deletion on ASFV attenuation and the induction of protection may be strain dependent and, in some cases, the deleted viruses exhibit a virulence phenotype indistinguishable from the parental virus. For example, the deletion of the NL (DP71L) gene from virulent strains completely attenuates the European E70 strain in animals but had no effect in two African ASFV strains [127,128,129]. Additionally, ASFV strains Malawi and Georgia are both attenuated by deletion of the thymidine kinase (TK) gene but only the TK-deleted Malawi virus was capable of inducing a protective immune response in inoculated animals [124]. 

Recent studies have demonstrated that multiple-gene mutants in ASFVs can variably affect viral immunogenicity. The multiple deletion of 6 members of MGF360 and 505 combined with the 9GL gene produced an attenuated Georgia ASFV strain with improved safety, but was unable to confer protection to animals when challenged with the virulent parental virus [130]. In contrast, the virulent Georgia isolate modified by deletion of the 9GL and DP96R/UK virulence factors showed improved safety and protection compared to the deletion of 9GL alone [131]. These results clearly demonstrated that the serial deletion of a second virulence factor might produce safer recombinant live attenuated ASFV-vaccines, thus opening hopes for future work.

The BA71 genotype I isolate with the CD2v/EP402R (HA) gene deleted [45] induced protection in pigs challenged with the homologous (parental) virulent ASFV BA71 strain and against the heterologous virulent genotype I E75, and genotype II Georgia07 ASFV strain [34]. Combining some of the different mutations so far described might yield a vaccine prototype with potential field applications.

#### 6.3.3. Recombinant LAVs Obtained from Attenuated Viruses

Several strategies to improve safety of attenuated strains (OURT88/3 or NH/P68) by deletion of several genes have provided variable results. The deletion of genes such as DP71L and DP96R (involved in virulence and clinical signs), or the A276R (an inhibitor of IFN), reduced the ability of the attenuated viruses to protect against challenge [132]. In contrast, some of these mutants showed a good degree of protection (60–100%) against challenge with the virulent strain Armenia 2007. In agreement with previous studies, however, the vaccine candidates induced (low) viremia and side-effects such as arthritis and necrotic foci in most of the vaccinated pigs [43,44], which would prevent their commercial use. The main antiviral response, type I IFN, is critical for the virus attenuation and induction of protection. However, it is critical to achieve a balance such that efficient viral replication occurs to induce an effective immune response but avoid clinical signs [44,130,131].

#### 6.3.4. Cell Lines for Production of LAVS

Primary porcine macrophage and monocyte culture systems are used in laboratories for biological and immunological studies of ASFV. However, primary cells would be unlikely to be used in vaccine production due batch to batch variations and the laborious and costly methods to obtain cells from animal donors. These issues were partially overcome by the adaptation of some ASFV isolates to grow in different stable monkey cell lines, such as Vero or MS cells, which have been routinely used for biological studies, production, and purification of the adapted virus [133,134,135]. However, the adaptation of ASF viruses has always resulted in genomic changes to the point of reducing the virus replication in pigs such that protection is not achieved [136]. In relation to this, five different porcine cell lines of monocyte origin have been developed so far: ZMAC, IPAM WT, IPAM-CD163, WSL, and CΔ2+ [137,138,139,140,141]. In addition, the COS-cell line has shown to be highly efficient to sustain the “in vitro” replication of ASF viruses with little apparent adaptation [135,142,143]. The genetically modified LAV, the BA71 CD2 produced in COS cells, yielded an effective vaccine able to confer homologous and heterologous protection. The COS cell line was successfully used for the generation of the LAV without significant genome changes [45]. However, some “in vivo” experimental studies based on the attenuated NH/P68 strain have shown that the LAV produced in COS cells were unable to maintain the capacity to confer protection [144]. Therefore, further evaluation studies are required for the potential use of COS cells in vaccine production. Furthermore, no studies have been published so far determining the behavior of ASFV strains generated from cell lines such as WSL and COS-1.

## 7. Development of DIVA Test

The application of a vaccine is dependent on the availability of an accompanying discriminatory test (DIVA test) allowing differentiation between vaccinated and infected animals. In order to ensure proper monitoring of the vaccination campaign and of its impact on disease evolution in a vaccinated pigs, the vaccine will need both a positive and a negative marker to reliably differentiate between vaccinated animals and those naturally infected. Reliable DIVA tests should therefore be considered in parallel to vaccine development and adapted to the context of vaccinating farmed (injected administration) and free-ranging populations of suids (oral administration).

These tests might be relatively easy to design for either subunit vaccines or LAVs. The latter could be based on those genes that are deleted to provide a DIVA test based on a negative marker. The negative marker (e.g., virulence or IFN inhibitor genes) would first need to be evaluated for induction of antibodies in the non-vaccinated, infected animals which would be absent from the vaccinated animals. A positive selection could be used since markers present in the genome of manipulated strains (BGal, BGus, others), facilitate the discrimination between vaccine and natural strains using molecular or serological methods.

## 8. ASFV Vaccine Candidates Likely to Be Available in the Shortest Term

From the currently available data on vaccine development, the LAVs seem the most promising candidates in the short-term. The solid protection so far demonstrated by a number of LAVs (up to 100%), the increased safety achieved by making multiple gene deletions together with their potential to confer solid cross-protection, support optimism about their potential for field implementation in the medium term. Table 2 summarizes the most promising LAV candidates for vaccine development based on existing data/knowledge. In spite of their experimental success, further research is needed to confirm their safety, DIVA-capabilities, and efficacy in long-term controlled experiments; an essential requisite to offer optimal LAVs.

Research performed to develop ASFV subunit vaccines suggest greater caution regarding their prompt commercial implementation. In contrast to their intrinsic safer nature and DIVA-potential, the protection levels afforded against ASFV experimental challenge have been poor when compared with LAVs. A continuous research effort focused both on antigen discovery and on better understanding the mechanisms involved in ASFV protection should succeed in the longer-term.

## 9. Expert Commentary

Progress on ASFV vaccine development requires further research on virus biology and virus–host interactions at all levels. Research gaps include transcriptome analysis to identify those virus genes that are transcribed at different stages of the replication cycle, and a better knowledge of the virus structural proteins, particularly those on the virion surface. In addition, better knowledge of virus entry mechanisms, including the cell receptor(s) on pig macrophages, will identify targets for vaccine development. Further definition of the functions of ASFV proteins, in particular those that inhibit the host’s defenses, is needed to optimize development of LAVs.

The mechanisms involved in immune protection against ASFV are still poorly characterized. Evidence indicates that adaptive immune responses required to protect against ASFV involve both serological and cellular immunity. Host immune and/or concomitant co-pathogen infection status appear to impact ASFV virulence. Research in the mechanisms by which the natural hosts, including bushpig and warthog, resist ASFV disease may help to identify the key actors involved in protection to develop ASFV vaccines. However, we should keep in mind that these African wild pigs belong to different species than domestic pigs and wild boar. Stocks of these wild African species of suids available for experimentation and therefore adapted to live in captive conditions are not available and zoological gardens are reluctant to provide individuals for animal experimentation. Therefore, building up collections of warthogs and bushpigs for vaccine research should be an instrumental preliminary step to investigate the mechanisms and processes involved in the natural protection of wild African pigs against ASFV.

Due to those constraints, some experts consider the efforts should be focused on pigs as the target species of interest. Preliminary in vitro tests are absolutely necessary (e.g., continued ability to replicate in pig macrophages for LAV). Identification of the correlates of immune protection in vitro as well as in vivo would help to evaluate the induction of protection against challenge and reduce unnecessary painful challenges with ASFV. Although not required for vaccine registration, knowledge of correlates of protection can also assist in monitoring vaccine efficacy and reduce the costs of vaccine testing.

For wild boar populations, potential candidate vaccines need to be immunogenic after oral administration and this will probably require a higher virus titer in the vaccine. In addition, the oral vaccine needs to be stable in the external environment to avoid losing potency when it is exposed to low and hot temperatures, sunshine, and other environmental factors. Another consideration is that the bait needs to be designed to allow uptake and attractiveness for animals of all ages and at different seasons. For a feasible oral immunization scheme, a suitable delivery device in the form of bait is needed, as it has been successfully experience with oral vaccination campaigns of wild boars against Classical Swine Fever [145,146].

### 9.1. Gaps and Future Directions for LAVs

Despite recent successes in the use of LAVs, there are still some important gaps and uncertainties that should be considered. LAVs have been demonstrated to confer solid protection (up to 100% surviving) against ASFV experimental challenge. However, the safety of ASF LAVs is of crucial importance. For vaccine registration, safety and immunogenicity has to be established over a range of doses and safety of repeated administration and overdose should be investigated.

It is also necessary to establish the genetic stability of LAVs during culture in vitro and pig passage in vivo. Lack of recombination in vaccination experiments with wild type challenge virus should be established. Multiple deletions in one single LAV candidate should reduce this possibility to the minimum. Since resources are limited and the costs of completing all experiments required for vaccine registration are very costly, it will be important to establish a pipeline for evaluation of LAV candidates which will enable early selection of the best candidates to be taken forward for further evaluation. Ideally this would include some selection at the stage of cell culture. Identifying correlates of in vitro pathogenesis and ability to induce protection is therefore important.

Other gaps are related to the selection of targeted virulence genes to be deleted. However, it is not always clear which virulence genes to target, since the effects of gene deletion on ASFV attenuation and protection can be strain dependent. Thus, testing gene deleted viruses from different genotypes may be needed to obtain strains that provide protection against isolates circulating in different regions. Further work is required to optimize the combinations of genes that can be deleted to produce a LAV that can meet safety standards required for registration and induce a good level of protection.

Another important issue unresolved is the availability of a licensed cell line to grow the LAVs for vaccine production. Some success using candidate established cell lines has been reported but more work is needed to optimize commercial productions systems.

### 9.2. Gaps and Future Directions for Subunit Vaccines

The subunit vaccine candidates also require further work. Several ASFV proteins have been associated with protection, but no specific viral protein(s) has been shown to be sufficient to confer full robust protective immunity in pigs. Thus, it is likely that additional antigens with protective potential will be identified and that a small pool of these that can induce high levels of protection will be defined. Previous studies [105] demonstrated that inactivated virions did not protect against infection. In addition, optimized delivery/vector systems are required to induce good levels of immune responses. Several immunization strategies and delivery/vector systems have been used to immunize pigs with a variety of different ASFV antigens. Results from these experiments are difficult to compare and interpret since the antigens or the delivery/vector system may not be optimal to induce a protective immune response. Further work is required to define protective antigens and to optimize delivery systems and strategies for vaccination to induce protective responses. Eventually, systems that can be applied in the field commercially will also need to be defined. Viral vectors that can deliver several antigens/genes should be investigated. Finally, the possibility of inducing “enhancing” antibody responses should be kept in mind.

### 9.3. Programs for Vaccine Candidate Evaluation

The development of new vaccines is dependent upon robust preclinical animal models in order to select those vaccine candidates which should progress to clinical development. The design of programmes to evaluate vaccine candidates in experimental conditions should be prepared according to the requirements of the European veterinary immunological legislation guidelines [http://www.ema.europa.eu/ema/index.jsp?curl=pages/regulation/general/general_content_000374.jsp&mid=WC0b01ac058002ddc5], taking into account the critical requirements for vaccine registration in the EU such as target species (swine, wild boar), categories (young/older animals, pregnant), routes of the vaccine administration, animal welfare for the in vivo experiments, vaccine dose (depending on vaccination schedule proposed), standardized clinical data collection and analysis (techniques, target samples, etc), routes of infection, challenge doses, challenge virus strain/s to be used, vaccination period and safety studies (including absence of reversion to virulence), and environmental risk assessment (capacity of the virus to survive, establish, and disseminate and pathogenicity to other live organisms).

The gaps identified mainly revolve around two aspects: (i) to harmonize and standardize clinical data collection and analysis and, (ii) to reduce the size, lengths, and costs of clinical trials. A critical deficiency is still the absence of a reliable “in vitro” correlation of “in vivo” protection. Thus, the only unarguable proof for an antigen or LAV to become a real vaccine candidate comes from its potential to clinically protect the target species (pigs and wild boar). This is considered one of the major challenges to progress in ASF vaccine development since there is no animal model other than swine. Safety experiments using LAVs need long experiments with a large number of animals. Vaccines are tested and selected using vaccination-challenge experiments in pigs which require strict biosafety level 3 (BSL3) animal facilities. Such procedures are not only extremely expensive, but are also environmentally and ethically difficult, considering the moderate to severe animal suffering associated with disease development, and the requirement that all animals be slaughtered at the end of the experimentation.

According to the European legislation, the guidelines for immunological veterinary medicines, following the in vivo laboratory tests, the efficacy and safety of the future vaccines should be evaluated by field trials. Field trials are a key point to evaluate the risk–benefit of vaccine candidates. Their design will depend on the characteristics of the vaccine (DIVA vaccine, formulation) and the infection under different virus–host scenarios (different virulence, different densities of hosts, and different durations of viremia and probabilities of developing chronic infections).

## 10. Conclusions

The recent alarming spread of ASF in Eastern Europe demands immediate countermeasures, with development of a vaccine as an important priority and as an additional good tool accompanying sanitary control measures.

Considerable progress has been made in the last decade leading to the development of ASF attenuated strains that have the potential to be used as candidate vaccines in a short/medium term. However, there are a number of important issues to clarify before a LAV is available for commercial development. Further in vivo testing with existing candidate LAVs to confirm acceptable levels of safety and efficacy against relevant field strains is a mandatory step and ensuring safety is curerntly the major challenge for its field implementation. More studies are needed to develop in vitro correlates of protection to facilitate selection of the most promising vaccine candidates and reduce the number of animal challenge experiments. In addition, for LAV production, identifying a suitable cell line is a priority.

The development of an efficient vaccine based on individual determinants from the ASFV (subunit vaccine) also requires parallel research efforts. In contrast to LAVs, subunit vaccines present the advantage of their innocuous nature, however, they would require a long-term effort in terms of research on protective antigen discovery and effective delivery mechanisms.

For the use of any type of vaccine in the field a DIVA test should be developed in parallel.

In principle, the same vaccine strain could be designed for domestic pigs and wild boar. Natural populations of wild boar have been effectively vaccinated against other infectious diseases through the use of LAVs administered orally by the distribution of palatable baits. However, the development of a specific vaccine to be successfully used in the wild boar will probably pose additional challenges related to vaccine administration route, efficacy, safety, its use in the environment, and monitoring immunity in natural populations. In both cases, domestic pig and wild boar, the vaccine should be orientated to give a response to the growing threat of ASF and directly combat the epidemic situation. For this purpose, field managing vaccination plans should be designed based on risk assessment strategies to effectively control the disease according to the different epidemiological scenarios present in Africa and Eastern Europe, and to reduce the threat and the possibility of ASF introduction into disease-free regions.

## Figures and Tables

**Table 1:** Approaches for development of subunit protein or DNA vaccines.

Genes/Proteins Delivered	Type of Vaccine	Challenge	Reference
p54/E183L, p30/CP204L	Baculovirus expressed proteins	Partial protection	[98]
P54/E183L, p30/CP204L, p72/B646L	Baculovirus expressed proteins	No protection	[100]
CD2v/pEP402R	Baculovirus expressed proteins	Partial protection	[62]
p54/E183L, p30/CP204L	DNA vaccination	No protection	[107,117]
Ubiquitin-CD2v/pEP402R- p54/E183L-p30/CP204L	DNA vaccination	Partial protection	[117]
DNA expression library	DNA vaccination	Partial protection	[108]

**Table 2:** Promising progress towards the development of a ASFV LAV.

Parental ASFV	Vaccine Type	ASFV Vaccine	Cell Production System	PROTECTION	References
NH/P68 (att)	Naturally attenuated	NHV/P68	PBM	Heterologous strain (L60, ARM07)	[44,63]
OURT88/3 (att)	Naturally attenuated	OURT88/3	BM	Homologous/ heterologous strain (OURT88/1, UG65)	[31,36,124]
Georgia07 (vir)	Genetically modified	Georgia07Δ9GL&DP96R/UK	PAM	Homologous strain	[126]
Ba71 (vir)	Genetically modified	Ba71ΔCD2/EP402R	COS	Homologous and heterologous strain (E75, GEORGIA07)	[88]
Benin (vir)	Genetically modified	BeninΔMGF	BM	Homologous strain	[130]
Benin (vir)	Genetically modified	BeninΔDP148R	BM	Homologous strain	[130]
NH/P68 (att)	Genetically modified	NH/P68ΔA238L	COS + 4 passages in PAM	Homologous and heterologous strain (ARM07)	[43]

Att = attenuated, Vir = virulent. Cell systems: Porcine blood monocyte/ macrophages (PBM), pig bone marrow cells (BM), monkey kidney tissue derived cells (COS), or porcine alveolar macrophages (PAM).
